# Hypertrophic Pulmonary Osteoarthropathy as an Initial Manifestation of Lung Cancer

**DOI:** 10.7759/cureus.98619

**Published:** 2025-12-07

**Authors:** Sarra K Mohamed Ahmed, Yara K Mohamedahmed, Lana Fadl, Dalia Salih, Razan K Mohamedahmed

**Affiliations:** 1 Internal Medicine, Walsall Manor Hospital, Walsall, GBR; 2 Internal Medicine, The James Cook University Hospital, Middlesbrough, GBR; 3 Internal Medicine, Walsall Healthcare NHS Trust, Walsall, GBR; 4 Geriatrics, Walsall Manor Hospital, Walsall, GBR; 5 Internal Medicine, Al-Adwani General Hospital, Taif, SAU

**Keywords:** digital clubbing, lung cancer, non-small cell lung carcinoma, paraneoplastic syndrome, rheumatoid arthritis mimic, unexplained arthralgia

## Abstract

Hypertrophic pulmonary osteoarthropathy (HPOA) is a paraneoplastic syndrome characterised by digital clubbing, periostitis, and arthralgia, most commonly secondary to lung malignancy. This case report presents a man in his 60s with a long history of smoking and asbestos exposure who developed progressive wrist and ankle pain, limb swelling, and enlargement of the hands and feet over eight months. He was initially investigated for rheumatoid arthritis due to positive rheumatoid factor (RF) and anti-citrullinated peptide (anti-CCP) antibodies, but repeated chest X-rays were normal. A CT scan later revealed a left lower lobe lesion, and biopsy confirmed squamous cell carcinoma of the lung. This case underscores the importance of maintaining a high index of suspicion for paraneoplastic syndromes such as HPOA when unexplained arthropathy and digital clubbing occur, even in the absence of respiratory symptoms.

## Introduction

Hypertrophic osteoarthropathy (HOA) is a syndrome characterised by periosteal new bone formation of the long bones, usually symmetrical, and commonly associated with digital clubbing and soft tissue changes. It may occur as a rare primary form (pachydermoperiostosis) or, more commonly, as secondary HOA, which represents 95-97% of cases [1,2]. Secondary HOA is most frequently linked to pulmonary disease, particularly bronchogenic carcinoma, and has therefore been termed hypertrophic pulmonary osteoarthropathy (HPOA) [3]. Pulmonary malignancies account for nearly 80% of malignancy-related cases, predominantly non-small cell types, with lung cancer responsible for over 60% of adult HOA and around 20% of isolated digital clubbing [4,5]. 

Clinically, HOA presents with pain, swelling, and tenderness of the long bones, along with rapidly progressive digital clubbing. In adults, up to 90% of cases are associated with intrathoracic malignancy or infection, while a smaller proportion arises from cardiovascular, gastrointestinal, hepatobiliary, or endocrine diseases [4,6]. Importantly, HOA may precede the detection of an underlying disease, most commonly a malignancy, particularly in patients aged 50-70 years, and its early recognition can facilitate timely diagnosis and management, thereby improving clinical outcomes.

## Case presentation

A man in his 60s with a 40-year history of smoking and asbestos exposure presented with an eight-month history of progressive ankle and wrist pain, joint stiffness, and increasing ring and shoe size. During this period, he was investigated by the rheumatology team for suspected inflammatory arthritis. His initial laboratory findings, including elevated erythrocyte sedimentation rate (ESR), positive rheumatoid factor (RF) and positive anti-citrullinated peptide (anti-CCP) antibodies, as well as antinuclear antibodies (ANA), are summarized in Table 1.

**Table 1:** Blood results from December 2024 and July 2025 with reference ranges ESR, erythrocyte sedimentation rate; RF, rheumatoid factor; anti-CCP, anti-citrullinated peptide; ANA, antinuclear antibodies; CRP, C-reactive protein

Investigation	Patient value in December 2024	Patient value in July 2025	Reference range
ESR	93 mm/hr	95 mm/hr	1-10 mm/hr
Anti-CCP	120 U/mL	128 U/mL	0-7 U/mL
CRP	122 mg/L	108 mg/L	0.0-5.0 mg/L
BNP	-	84 pg/mL	<100 pg/mL
RF	85 IU/mL	79 IU/mL	<14 IU/mL

Despite treatment with non-steroidal anti-inflammatory drugs (NSAIDs) and a short course of steroids, his symptoms persisted and progressively worsened.

**Figure 1:**
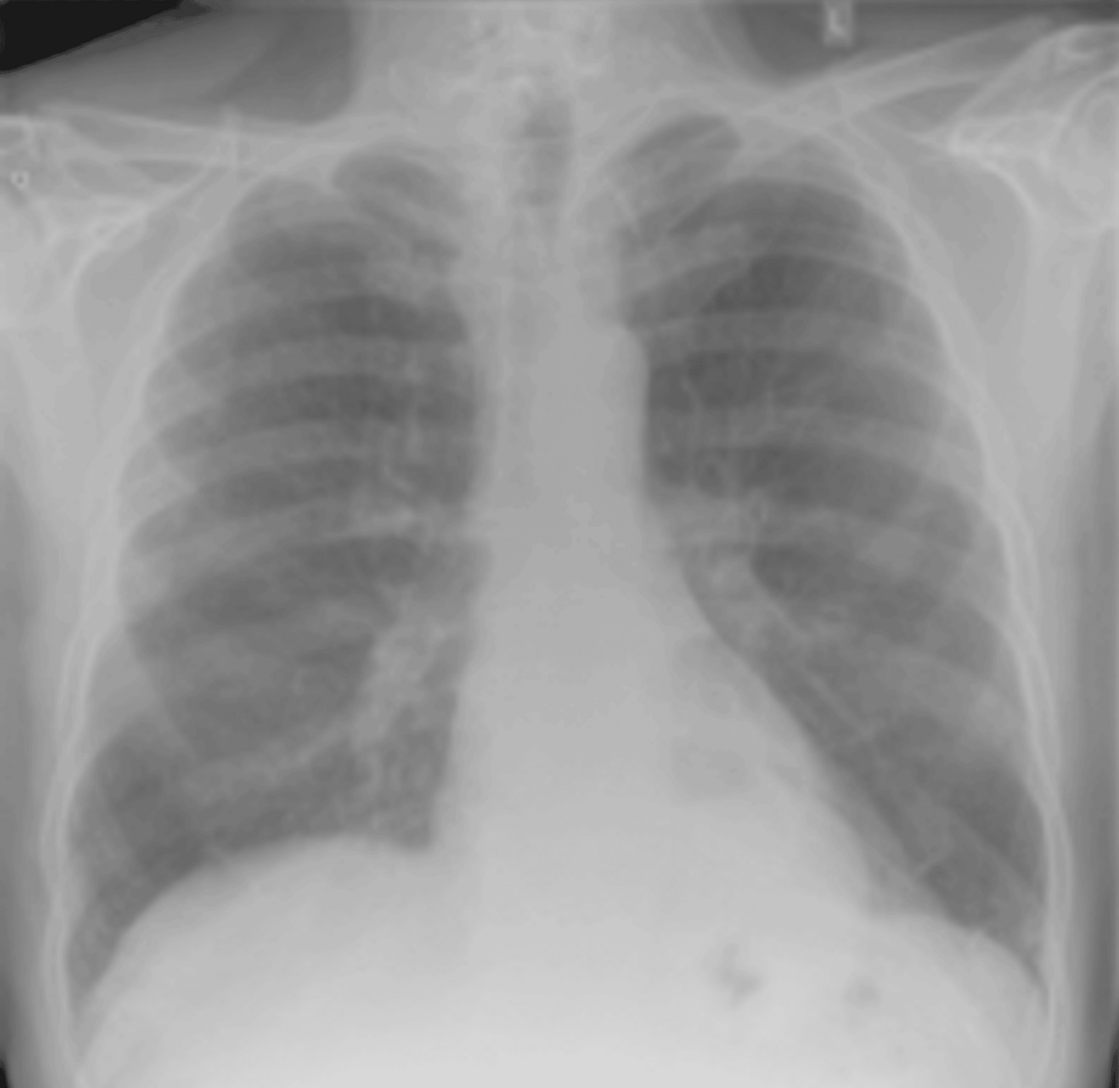
Chest X-ray with normal findings

Six weeks prior to his current presentation, he developed a new dry cough, a one-stone weight loss over eight weeks, lower-limb swelling, and intermittent night sweats. He attended the same-day emergency care (SDEC) clinic, where initial suspicion was for possible heart failure. However, B-type natriuretic peptide (BNP) was only 84 pg/mL, making this less likely. Repeat laboratory testing showed an ESR of 95 mm/hr, and the chest X-ray remained normal (Figure 1).

On examination, he was comfortable at rest with an oxygen saturation of 96% on room air. There was pitting edema up to the mid-shin, marked digital clubbing, and tenderness of the wrists and ankles. Bilateral Dupuytren contractures were also noted. Chest auscultation demonstrated increased vocal resonance in the middle left lung zone with a positive whispered pectoriloquy, raising the suspicion of an underlying pulmonary malignancy.

**Figure 2:**
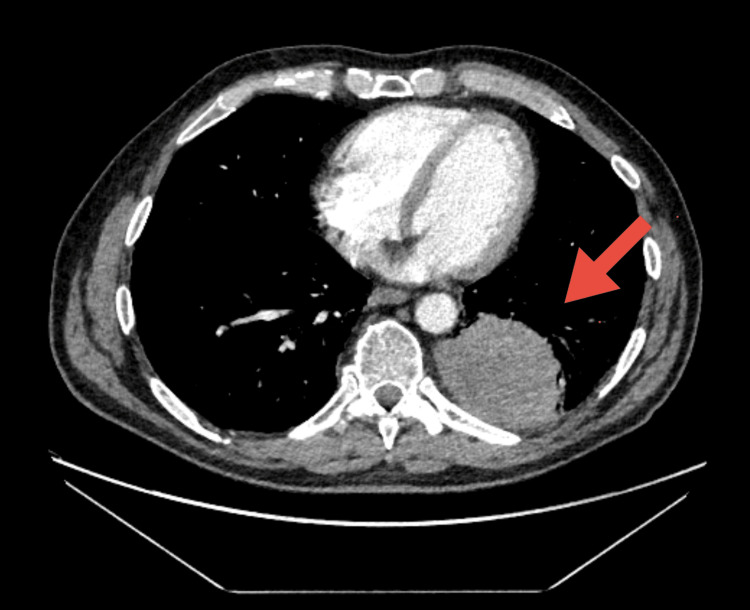
CT thorax showing a 2.8 cm left lower lobe mass (arrow) abutting the posterior pleura

A CT scan of the thorax, abdomen, and pelvis was performed, demonstrating a 2.8 cm left lower-lobe lesion abutting the posterior pleura with mediastinal and hilar lymphadenopathy and omental lymph node involvement, consistent with primary lung cancer with nodal disease (Figure 2).

The patient was referred to the lung multidisciplinary team (MDT) for further evaluation. As bronchoscopy was not tolerated, a CT-guided biopsy was performed, confirming a diagnosis of squamous cell carcinoma of the lung.

## Discussion

HPOA is a paraneoplastic musculoskeletal syndrome most often secondary to intrathoracic disease, particularly non-small cell lung carcinoma (NSCLC), including both adenocarcinoma and squamous cell carcinoma [1,3]. The condition is characterised by digital clubbing, periostitis of long bones, and arthralgia or joint effusion, typically bilateral and symmetrical [3,4]. Secondary forms account for over 95% of all cases, and pulmonary malignancies represent approximately 80% of malignancy-related HPOA [1,4,5].

In this case, the patient’s arthralgia and enlargement of the hands and feet preceded the development of respiratory symptoms by several months. Despite multiple normal chest X-rays, no pulmonary lesion was identified until a CT scan revealed the underlying malignancy. This highlights that HPOA may clinically manifest long before respiratory or radiological evidence of lung cancer, underscoring the importance of clinical suspicion in high-risk individuals such as long-term smokers or those with asbestos exposure [1,2,4].

The diagnostic challenge in this case closely mirrors that described by Shekar et al. (2023), wherein arthralgia was the presenting symptom of NSCLC-associated HPOA in a patient with rheumatoid arthritis, leading to an initial misdiagnosis [2]. Similarly, our patient’s positive RF and anti-CCP antibodies initially suggested rheumatoid arthritis, resulting in a delay in identifying the true cause. However, the absence of morning stiffness, sparing of small hand joints, and a non-inflammatory pattern of pain were inconsistent with classic RA, suggesting a paraneoplastic process instead.

Although anti-CCP antibodies have a specificity of up to 98% for rheumatoid arthritis, they may be falsely elevated in patients with lung cancer [6,7]. Baka et al. (2011) found that 5.9% of patients with lung cancer had raised anti-CCP antibodies, possibly due to citrullinated protein formation within malignant lung tissue, enhanced by smoking-related inflammation [6]. Larson et al. (2011) similarly reported anti-CCP-positive paraneoplastic polyarthritis, reinforcing that tumor-driven immune activation can mimic autoimmune disease [7].

This case emphasizes that persistent or atypical arthropathy, particularly when serological findings do not align with clinical features, should prompt consideration of paraneoplastic syndromes such as HPOA. Even in the absence of respiratory symptoms or radiographic abnormalities, CT imaging should be pursued when the clinical picture remains suspicious. Early recognition of HPOA can enable timely lung cancer diagnosis and improve patient outcomes [1,2,4].

## Conclusions

This case highlights the importance of maintaining a high index of suspicion for hypertrophic pulmonary osteoarthropathy as a paraneoplastic manifestation of lung cancer, even in the absence of respiratory symptoms or abnormalities on chest X-rays. In patients with unexplained arthropathy, digital clubbing, or atypical serological findings, particularly those with significant smoking or asbestos exposure, early use of CT imaging is essential. Prompt recognition of HPOA can lead to earlier cancer diagnosis and may improve clinical outcomes.
